# Percutaneous radiofrequency ablation of an actively bleeding renal angiomyolipoma

**DOI:** 10.31744/einstein_journal/2024RC1008

**Published:** 2024-11-22

**Authors:** Pedro Ivo Ravizzini, Henrique Augusto Lino, Gregory Ravizzini, Luís Gustavo Morato de Toledo

**Affiliations:** 1 Faculdade de Ciências Médicas da Santa Casa de São Paulo Radiology Department São Paulo SP Brazil Radiology Department, Faculdade de Ciências Médicas da Santa Casa de São Paulo, São Paulo, SP, Brazil.; 2 Faculdade de Ciências Médicas da Santa Casa de São Paulo Urology Department São Paulo SP Brazil Urology Department, Faculdade de Ciências Médicas da Santa Casa de São Paulo, São Paulo, SP, Brazil.; 3 Universidade de São Paulo Hospital das Clínicas São Paulo SP Brazil Hospital das Clínicas, Universidade de São Paulo, São Paulo, SP, Brazil.; 4 MD Anderson Cancer Center Houston TX United States MD Anderson Cancer Center, Houston, TX, United States.

**Keywords:** Radiofrequency ablation, Catheter ablation, Angiomyolipoma, Kidney neoplasms, Hemorrhage, X-ray computed tomography

## Abstract

We report a case of computed tomography-guided percutaneous radiofrequency ablation of a bleeding renal angiomyolipoma. Radiofrequency ablation was performed as an alternative to partial nephrectomy and super-selective renal artery embolization for ruptured renal angiomyolipoma with slow persistent bleeding in a patient with elevated serum creatinine levels and other comorbidities. Computed tomography-guided radiofrequency ablation successfully stopped the active hemorrhage and did not affect long-term renal function during the 3-year follow period. No complications were associated with the procedure. Radiofrequency ablation of a bleeding renal angiomyolipoma may be considered a more invasive surrogate procedure in an urgent setting; however, further studies are necessary to evaluate the long-term benefits of this approach and its overall impact on renal function compared to traditional methods.

## INTRODUCTION

Renal angiomyolipomas (AML) are usually benign solid tumors;^(1,2)^ however, they have an increased risk of spontaneous hemorrhage when >4 cm. Treatment options include super-selective renal artery embolization (SRAE), partial nephrectomy (PN), and radical nephrectomy (RN). While widely available, PN and RN are associated with substantial morbidity, and SRAE may still negatively impact renal function to varying degrees.^(1,3)^

We report the case of a patient presenting with acute hemorrhage due to a ruptured right renal AML, followed by slow and persistent bleeding, who was successfully treated with computed tomography (CT)-guided percutaneous radiofrequency ablation (RFA).

## CASE REPORT

A 76-year-old man with a 7-year history of biological aortic valve replacement, a definitive pacemaker for complete atrioventricular block, and prophylactic chronic warfarin anticoagulation presented to the Emergency Department with severe back pain, sweating, and malaise. The patient reported pain upon palpation of the right flank and tenderness of the right costovertebral angle. His blood pressure was 85/60 mmHg, and his heart rate was 73 beats per minute. Initial laboratory tests revealed a serum creatinine of 1.45 mg/dL (reference range: 0.5–1 mg/dL).

**Figure 1:**
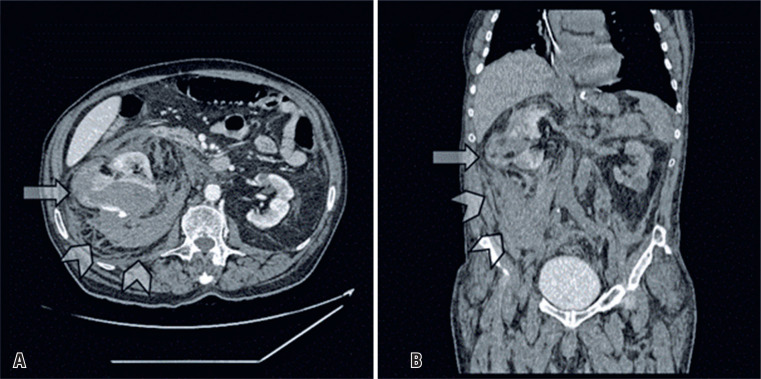
Axial (A) and the coronal (B) contrast-enhanced computed tomography images (soft tissues window) at admission showing an exophytic renal mass with a heterogeneous aspect, permeative septa, and fat-containing areas (white arrow) on the mid-pole of the right kidney, associated with perinephric and pararenal hematomas, and signs of inflammation of the adjacent fat (white arrowheads)

Contrast-enhanced abdominal CT showed an actively bleeding exophytic right cortical renal mass associated with a substantial retroperitoneal hemorrhage (Figure 1). Notably, mild bilateral renal cortical atrophy implied decreased baseline renal function. Warfarin was withheld, and even though his hemodynamic status remained stable, deterioration of his hemoglobin levels required transfer to the intensive care unit, where he received vitamin K, plasma, and blood transfusions. Despite conservative measures, a further decrease in his hemoglobin levels warranted prompt intervention. SRAE was initially considered; however, possible shortcomings, such as inadequate control of bleeding due to poor individualization of feeding arteries, unwanted embolization of healthy renal parenchyma, and excessive use of iodinated intravascular contrast media, leading to further compromise of renal function, were of concern. Partial nephrectomy was also considered; however, owing to the patient's comorbidities, the option was discarded, especially because of the risk of uncontrollable bleeding and conversion to RN.^(4)^ Considering the risks and benefits, we proposed CT-guided RFA as a minimally invasive treatment option for hemorrhage control with potential nephron-sparing benefits.

**Figure 2:**
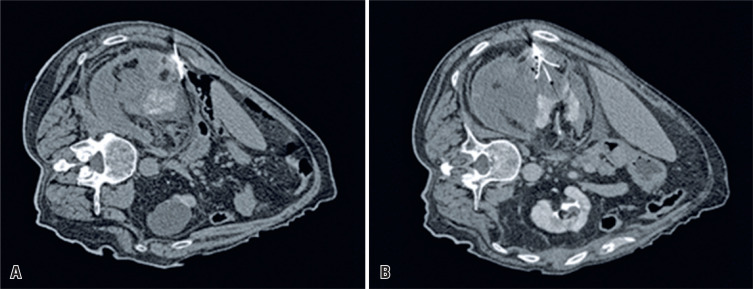
Sequential intraprocedural axial computed tomography images. (A) Hydro-dissection of the anterior surface of the right kidney with the radiofrequency ablation probe placed in the deep interface of angiomyolipomas and renal parenchyma; (B) Confirmation of the position of probe lines and radiofrequency ablation

The procedure was performed with the patient in the left lateral decubitus position to provide adequate access to the right kidney. Hydro-dissection was performed on the anterior surface of the right kidney using an 18 G Chiba needle (Argon Medical Devices, Rochester, NY, USA). Intravenous sodium bicarbonate was administered to help protect renal function, and 60 cc of non-ionic iodine contrast was used to delineate and clearly define the size and interface between the AML and renal parenchyma. A 25cm, 14 G RFA probe (Starburst Semi-flex-Angiodynamics/RITA system, Queensbury, New York, USA) was inserted under combined CT and ultrasound guidance and placed perfectly on the deep margin of the AML, ensuring that the probe tines extended into the normal renal parenchyma. Probe tines were deployed to create an ablation zone approximately 5 mm beyond the AML margin. The RFA probe was attached to a RITA model 1500X RF automatic generator (Angiodynamics/RITA system, Queensbury, New York), modulated for an average temperature of 221°F, as measured within the probe array. The base of the nodule was treated for two cycles, followed by subsequent retractions of approximately 1 cm and overlapping ablation zones until there were no areas of the AML left untreated (Figure 2). The time of individual ablations was impedance-controlled, depending on the tissue resistance and final confirmation of local temperatures >158 ^o^F. The probe was progressively removed through careful ablation of the insertion tract.

**Figure 3:**
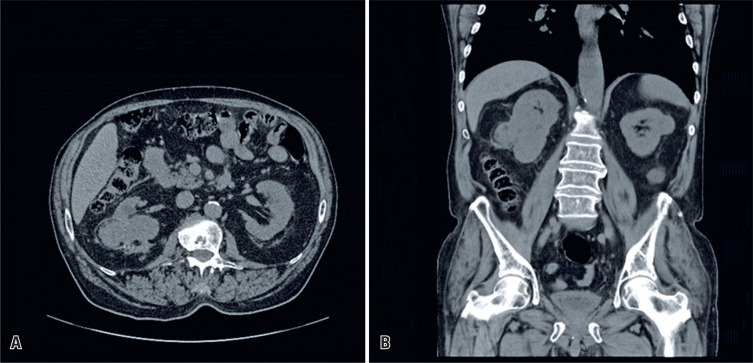
Axial (A) and coronal (B) non-enhanced computed tomography images (Soft tissues window) showing the size reduction in the angiomyolipomas after 3 months

Two days after the procedure, follow-up CT revealed no signs of active bleeding and a considerable reduction in the retroperitoneal hematoma. Approximately 3 months later, his creatinine levels reached 1.2 mg/dL and remained stable until the last follow-up 36 months after the procedure. CT images obtained at 3 months (Figure 3), 1 year, and 3 years showed a progressive reduction in AML.

This study has been approved by *Faculdade de* *Ciências Médicas da Santa Casa de São Paulo* (CAAE: 82934024.8.0000.5479, # 7.156.594).

## DISCUSSION

Spontaneous rupture is a rare and life-threatening complication of renal AMLs with variable outcomes and is directly associated with the rate and volume of hemorrhage during presentation.^(4)^ While sometimes unavoidable, PN and RN have relatively high morbidity rates, and SRAE may negatively impact renal function to various degrees.^(3–5)^

Radiofrequency ablation is an emerging alternative to surgical and endovascular procedures^(4)^ with relatively good success.^(6)^ The first description of RFA in an elective setting was provided by Prevoo et al.^(7)^ Further data from Castle et al^(4)^ showed that CT-guided RFA for the treatment of AML is a rapid, minimally invasive procedure with little or no complications, offering early hospital discharge. Moreover, this technique did not appear to promote renal function deterioration. Although there was no long-term follow-up to evaluate the recurrence of renal AML after RFA, their results may suggest overall lower healthcare costs.

Sooriakumaran et al^(3)^ followed up 102 patients with renal AML for approximately 10 years. Most patients selected for treatment underwent SRAE for various complications, such as post-embolization syndrome, abscess formation, and chronic renal failure. In contrast, temporary abdominal pain and hypoesthesia of the L1 dermatome were the only postprocedural complications described after RFA for AML.^(3)^ Some patients in the SRAE group required additional procedures for lesion recurrence, lack of size reduction, or rebleeding, in contrast to the RFA group, which showed no need for re-ablation.

Further studies are needed to analyze the long-term recurrence and rebleeding rates, morbidity and mortality indices, and the benefits of renal function preservation. Treatment success may be influenced by the size and location of AML within the kidney. Therefore, RFA indications may depend on individual patient selection but may be an option for PN and SRAE, particularly in the absence of an experienced vascular interventional radiologist.

## CONCLUSION

Percutaneous CT-guided radiofrequency ablation of a bleeding kidney angiomyolipoma may be considered in selected cases where patients are relatively hemodynamically stable. This technique may also offer nephron-sparing advantages as a viable option for nephrectomy. Further studies are required to evaluate the effectiveness, safety, and long-term benefits of radiofrequency ablation for bleeding renal angiomyolipomas.
